# Non-viral Gene Delivery of Interleukin-1 Receptor Antagonist Using Collagen-Hydroxyapatite Scaffold Protects Rat BM-MSCs From IL-1β-Mediated Inhibition of Osteogenesis

**DOI:** 10.3389/fbioe.2020.582012

**Published:** 2020-10-06

**Authors:** William A. Lackington, Maria Antonia Gomez-Sierra, Arlyng González-Vázquez, Fergal J. O’Brien, Martin J. Stoddart, Keith Thompson

**Affiliations:** ^1^AO Research Institute Davos, AO Foundation, Davos, Switzerland; ^2^Tissue Engineering Research Group, Department of Anatomy and Regenerative Medicine, Royal College of Surgeons in Ireland, University of Medicine and Health Sciences, Dublin, Ireland; ^3^AMBER Centre, Royal College of Surgeons in Ireland, Dublin, Ireland

**Keywords:** non-viral gene delivery, scaffold, osteogenesis, immunomodulation, IL-1Ra

## Abstract

Although most bone fractures typically heal without complications, a small proportion of patients (≤10%) experience delayed healing or potential progression to non-union. Interleukin-1 (IL-1β) plays a crucial role in fracture healing as an early driver of inflammation. However, the effects of IL-1β can impede the healing process if they persist long after the establishment of a fracture hematoma, making it a promising target for novel therapies. Accordingly, the overall objective of this study was to develop a novel gene-based therapy that mitigates the negative effects of IL-1β-driven inflammation while providing a structural template for new bone formation. A collagen-hydroxyapatite scaffold (CHA) was used as a platform for the delivery of nanoparticles composed of pDNA, encoding for IL-1 receptor antagonist (IL-1Ra), complexed to the robust non-viral gene delivery vector, polyethyleneimine (PEI). Utilizing pDNA encoding for Gaussia luciferase and GFP as reporter genes, we found that PEI-pDNA nanoparticles induced a transient gene expression profile in rat bone marrow-derived mesenchymal stromal cells (BM-MSCs), with a transfection efficiency of 14.8 ± 1.8% in 2D. BM-MSC viability was significantly affected by PEI-pDNA nanoparticles as evaluated using CellTiter Blue; however, after 10 days in culture this effect was negligible. Transfection with PEI-pIL-1Ra nanoparticles led to functional IL-1Ra production, capable of antagonizing IL-1β-induced expression of secreted embryonic alkaline phosphatase from HEK-Blue-IL-1β reporter cells. Sustained treatment with IL-1β (0.1, 1, and 10 ng/ml) had a dose-dependent negative effect on BM-MSC osteogenesis, both in terms of gene expression (*Alpl* and *Ibsp*) and calcium deposition. BM-MSCs transfected with PEI-IL-1Ra nanoparticles were found to be capable of overcoming the inhibitory effects of sustained IL-1β (1 ng/ml) treatments on *in vitro* osteogenesis. Ultimately, IL-1Ra gene-activated CHA scaffolds supported mineralization of BM-MSCs under chronic inflammatory conditions *in vitro*, demonstrating potential for future therapeutic applications *in vivo*.

## Introduction

In recent years, increasing significance has been given to the immune system and its role in determining the success of intrinsic tissue repair mechanisms. Following bone injury, inflammatory cells, including neutrophils and macrophages, initially debride the injury site and stimulate the formation of the fracture hematoma through the upregulated secretion of pro-inflammatory cytokines such as interleukin-1β (IL-1β) (Einhorn et al., 1995; Lackington and Thompson, 2020). A fracture hematoma is established to serve as a transient matrix for the recruitment of progenitor cells, such as bone marrow-derived mesenchymal stromal cells (BM-MSCs), and the deposition of new tissue (Schell et al., 2017). However, prolonged inflammation, lasting until after the fracture hematoma is established, can inhibit osteogenic differentiation of BM-MSCs and induce cell apoptosis, resulting in reduced bone volume and diminished mechanical strength (Guo et al., 2008; Reinke et al., 2013; Santolini et al., 2015; Sun et al., 2017). Taken together, prolonged inflammation can impinge fracture healing and contribute to instances of delayed healing and potential progression to non-union, which is observed in up to 10% of patients with fractures (Einhorn, 1998; Pountos et al., 2008). Thus, while necessary to initiate the intrinsic healing response to bone fracture, there is a requirement that these signals must then be tightly regulated for the microenvironment to become permissive to successful bone formation. Accordingly, immunomodulation of the fracture microenvironment could be a novel and promising way to overcome the detrimental effects of prolonged inflammation and improve bone healing.

Interleukin-1 receptor antagonist (IL-1Ra) is the natural antagonist of IL-1β, that could potentially serve as the basis of an immunomodulatory therapy for bone repair. There has been limited development of IL-1Ra-based therapies for bone repair. However, by virtue of its anti-inflammatory properties, IL-1Ra has formed the basis of a multitude of therapies to enhance BM-MSC chondrogenesis under IL-1β-driven inflammatory conditions (Glass et al., 2014), to promote articular cartilage repair pre-clinically (Zhang et al., 2015), and to treat rheumatoid arthritis clinically (Mertens and Singh, 2009). Anakinra, a recombinant IL-1Ra-based therapy, has been FDA approved since 2001 to reduce inflammation in the joints of patients with rheumatoid arthritis (Kay and Calabrese, 2004). Despite frequently reported dose-related side effects with Anakinra (Zeft et al., 2009), its effectiveness at reducing inflammation has been deemed to outweigh its off-target effects. More recently, it has been demonstrated that administration of IL-1Ra, using a fibrin matrix as a platform for local delivery, can enhance bone formation in critically sized calvarial defects of mice (Martino et al., 2016). Remarkably, it was shown that fibrin matrix loaded with recombinant IL-1Ra performed significantly better compared to fibrin matrix loaded with BM-MSCs, which marks a commonly proposed therapy for bone repair (Martino et al., 2016).

This study proposes a novel IL-1Ra gene-based therapy based on the existing technology and proven regenerative potential of collagen-hydroxyapatite (CHA) scaffolds (Cunniffe et al., 2010a; Curtin et al., 2012). The novel therapy combines the proven regenerative capacity of collagen-based scaffolds as templates for new tissue formation (Lyons et al., 2010; Cipitria et al., 2012), with the versatility of non-viral gene delivery vectors as a method to locally deliver IL-1Ra (Fernandes et al., 1999). An IL-1Ra gene-activated collagen-based scaffold provides a cost-effective method to locally administer IL-1Ra gene therapy for long bone repair, with several key advantages compared to recombinant protein therapy. Expression levels of IL-1Ra can remain therapeutically effective while keeping below supraphysiological levels that are typically used in recombinant protein-based therapies, greatly reducing the risk of off-target effects. Transient gene expression permits that the initial inflammatory response to injury can be better preserved, as opposed to recombinant protein-based therapies that typically exert a burst release of drug after administration. Lastly, the stability of gene-based therapies facilitates an ‘off-the-shelf’ approach, compared to protein-based therapies, which suffer from issues associated with protein degradation. Polyethyleneimine (PEI), a cationic polymer, was selected for IL-1Ra gene delivery due of its established potential as a non-viral gene delivery vector. Our team has successfully used PEI previously in combination with collagen-based scaffolds for enhancing their regenerative capacity when complexed to BMP-2 and VEGF for applications in bone repair (Tierney et al., 2012; Curtin et al., 2015), and NGF, GDNF and c-Jun for applications in peripheral nerve repair (Lackington et al., 2018). Thus, the overall objective of this study was to develop an IL-1ra gene-activated CHA scaffold for application in long bone repair.

Specifically, this study aimed (1) to determine the transfection efficiency and transgene expression profile of BM-MSCs using Green Fluorescent Protein (pGFP) and *Gaussia* Luciferase (pGLuc) as reporter genes; (2) to assess the bioactivity of PEI-pIL-1Ra nanoparticles using HEK-Blue-IL-1β reporter cells, (3) to evaluate the inhibitory effects of IL-1β on BM-MSC osteogenesis in 2D in terms of gene expression and phenotype, and (4) to assess the bioactivity of IL-1Ra gene delivery on protecting the osteogenic phenotype of BM-MSCs cultured under IL-1β-driven inflammatory conditions in 2D, and in a CHA scaffold.

## Materials and Methods

### PEI-pDNA Nanoparticles Formulation

#### Preparation and Propagation of Plasmid DNA

Plasmid DNA (pDNA) encoding *Gaussia* Luciferase (pGLuc) was purchased from New England Biolabs (Ipswich, MA, United States) and used as a reporter gene to determine the transgene expression profile in transfected BM-MSCs. pDNA encoding for GFP (pGFP) was purchased from Lonza (Cologne, Germany) and used to determine the transfection efficiency of PEI-pDNA nanoparticles. pDNA encoding for IL-1Ra (pIL-1Ra, accession number XM_006233636.3) was cloned into the multiple cloning site (MCS) of pcDNA3.1(+)-C-eGFP plasmid (GenScript, Piscataway, NJ, United States). The plasmid contains the DNA sequence for reporter GFP downstream of the MCS, thereby allowing visualization of transfection efficiency. All pDNA constructs were propagated by transforming OneShot TOP10 chemically competent *E. coli* (Invitrogen, Switzerland) according to the manufacturer’s protocol. The pDNA was purified using Endofree plasmid maxi kit (Qiagen, Switzerland) as per the manufacturer’s instructions. Purified pDNA was then quantified using a Nanodrop 1000 spectrophotometer, and the plasmids were diluted and used at a concentration of 0.5 μg/μl in Tris-EDTA buffer (pH 8) (Sigma-Aldrich, Switzerland).

#### Complexation of pDNA to PEI

Nanoparticles were formulated by complexation for 30 min between the cationic gene delivery vector and a constant amount of anionic pDNA (pGLuc, pGFP, or pIL-1Ra). Branched 25 kDa polyethyleneimine (Sigma-Aldrich) was selected as a gene delivery vector in this study due to its previously reported applications in combination with collagen-based scaffolds (Tierney et al., 2012; Curtin et al., 2015; Lackington et al., 2018). PEI was used at a working concentration of 0.1 mg/ml, and mixed with 2 μg of pDNA in a reaction volume of 50 μl so that the resulting ratio between the moles in PEI amine groups and the moles in pDNA phosphate groups (N/P) was equal to 7.

### Characterization of BM-MSC Transfection With PEI-pDNA Nanoparticles

#### Culture of Rat Bone Marrow-Derived BM-MSCs

BM-MSCs were targeted in this study given their high presence at a fracture site, with the assurance that they can migrate through the porous microarchitecture of an implanted CHA scaffold (Castano et al., 2015; Raftery et al., 2017; Walsh et al., 2018). Fischer 344 rat BM-MSCs were purchased from Cyagen Biosciences (Jiangsu, China). BM-MSCs were expanded on tissue culture plastic with expansion medium defined as minimum essential media α (Gibco, Switzerland), supplemented with 10% fetal bovine serum (FBS) (Corning, Switzerland), 100 U/ml penicillin, 100 μg/mL streptomycin (both Gibco) and 10 ng/ml basic fibroblast growth factor (Sigma-Aldrich). BM-MSCs were expanded and passaged at an initial cell seeding density of 3000 cells/cm^2^. To induce osteogenesis, BM-MSCs were cultured in osteogenic media (OM) composed of DMEM (1 g/L glucose), supplemented with 10% FBS, penicillin-streptomycin, 10 nM dexamethasone (Sigma-Aldrich), 50 μg/mL ascorbic acid-2-phosphate (Sigma-Aldrich), and 5 mM β-glycerol phosphate (Sigma-Aldrich). Basal media, used as a control in some assays, was composed of DMEM (1 g/L glucose), supplemented with 10% FBS and penicillin-streptomycin.

#### Transfection of BM-MSCs

Prior to transfection, MSCs were seeded at a density of 50,000 cells/well in 6-well plates (Corning, Fisher Scientific, Switzerland) and allowed to grow in expansion medium for 24 h. Expansion medium was then removed, cells were washed with PBS and then incubated in reduced serum OptiMEM (Gibco, Switzerland) for 1 h. Nanoparticles were formulated as described and suspended in a total volume of 500 μL OptiMEM, which was then added to each well to initiate transfection. After 4 h, transfection media was removed, cells were washed with PBS and expansion medium was added to each well.

#### Evaluation of Transfection Efficiency

The reporter gene pGFP was used to determine the transfection efficiency of BM-MSCs. BM-MSCs were transfected with PEI-pGFP nanoparticles and the expression of GFP was assessed at 3, 7, and 14 days post-transfection using an EVOS fluorescence microscope (Thermo Scientific, Switzerland). Cells exhibiting green fluorescence were deemed to be successfully transfected. The transfection efficiency at each time-point was evaluated by determining the percentage of GFP^+^ cells in a population of transfected cells, following detachment with trypsin-EDTA solution (Gibco), using flow cytometry. Data was acquired on a FACS Aria III flow cytometer (BD Biosciences) and analysis was performed using BD FACS Diva software (BD Biosciences).

#### Evaluation of Transgene Expression Profile

The reporter gene pGLuc was used to determine the transgene expression profile in transfected cells. BM-MSCs were transfected with PEI-pGLuc nanoparticles and samples of media were collected from transfected cells at 3, 7, 10, and 14 days after transfection. Luciferase content in collected media was assessed using the Pierce Gaussia Luciferase Flash Assay Kit (Thermo Scientific). Luciferase assay solution was prepared by diluting 100X coelenterazine stock to 1X working concentration in Gaussia Flash Assay Buffer. The assay was carried out in opaque 96-well plates by mixing 20 μL of sample with 50 μL of 1X luciferase assay solution. Luminescence in each well was recorded in terms of relative luciferase units using a Victor 3 plate reader (PerkinElmer, United States).

#### Assessment of PEI-pDNA Nanoparticle Cytotoxicity

To evaluate the effect of PEI-pDNA nanoparticles on BM-MSC cytotoxicity, CellTiter-Blue Cell Viability Assay (Promega, Switzerland) was carried out at 3, 7, 10, and 14 days post-transfection. Transfected cells were washed with PBS, and expansion medium containing the CellTiter-Blue reagent was added as per the manufacturer’s instructions. Cells were incubated for 4 h and the fluorescence (excitation 560 nm; emission 590 nm) was determined using a Victor 3 plate reader.

#### Assessment of PEI-pIL-1Ra Nanoparticle Bioactivity

HEK-Blue IL-1β cells (Invivogen, Switzerland) were used to assess the bioactivity of the gene product after transfection with PEI-pIL-1Ra nanoparticles. This cell line detects IL-1β-mediated activation of NF-κB and AP-1 pathways, which drives production of the secreted embryonic alkaline phosphatase (SEAP) reporter. The cell line was transfected with PEI-pIL-1Ra nanoparticles as described in Section “Transfection of BM-MSCs” and grown using DMEM (4.5 g/l glucose), 2 mM L-Glutamine, 10% FBS, 100 U/ml penicillin, 100 μg/ml streptomycin, 100 μg/ml Normocin. To determine whether HEK-Blue IL-1β cells were successfully transfected with PEI-pIL-1Ra nanoparticles, the expression of GFP was assessed 3 days post-transfection using an EVOS fluorescence microscope. Non-transfected cells were then treated for 24 h with recombinant rat IL-1β (30 ng/ml) (R&D Systems, United States) alone or in combination with recombinant rat IL-1Ra (100 ng/ml) (R&D Systems). In parallel, cells transfected with PEI-pIL-1Ra were also treated with IL-1β (30 ng/ml) to test whether the gene product could inhibit the induction of SEAP. Conditioned medium was collected after 24 h of treatment with IL-1β, and SEAP levels in the samples were quantified using QUANTI-Blue reagent, according to the manufacturer’s instructions (Invivogen, Switzerland).

#### Assessment of IL-1Ra Produced by Rat BM-MSCs Transfected With PEI-pIL-1Ra Nanoparticles

Rat BM-MSCs were transfected with PEI-pIL-1Ra nanoparticles as described in Section “Transfection of BM-MSCs.” Culture media from transfected and non-transfected cells was collected at 3, 7, 10, and 14 days post-transfection. The levels of IL-1Ra in collected media were determined by ELISA (R&D Systems, Switzerland). At the same timepoints, the amount of dsDNA in cultures was determined using PicoGreen dsDNA assay (Invivogen, Switzerland), in order to normalize the quantity of IL-1Ra produced by cells (pg) to the quantity of dsDNA found in cultures (μg).

### Assessment of the Capacity of IL-1Ra Gene Delivery to Protect the Osteogenic Phenotype of BM-MSCs Cultured Under Inflammatory Conditions

#### Evaluation of the Effect of Sustained IL-1β Treatment on Osteogenic Gene Expression

Expression of osteogenic markers: alkaline phosphatase (*Alpl*) and integrin binding sialoprotein (*Ibsp*), was then assessed in BM-MSCs, following treatment with IL-1β. BM-MSCs were cultured using osteogenic medium (OM) as described in Section “Culture of Rat Bone Marrow-Derived BM-MSCs,” and treated with either 0.1, 1, or 10 ng/ml IL-1β for the duration of the culture period, with medium changed three times per week. RNA was isolated from cells after 3, 7, and 14 days in culture using RNeasy mini kit columns (Qiagen, Switzerland), and RNA quality was checked by assessing A260/280 and A260/230 values while determining RNA concentration using a Nanodrop 1000 Spectrophotometer (Thermo Scientific). Subsequently, 500 ng of RNA was used to generate cDNA samples by TaqMan-mediated reverse transcription (Applied Biosystems, United States). Reverse transcription mastermix included 10X TaqMan RT buffer, 25 mM Magnesium Chloride, dNTPs, random hexamers and RNase inhibitor (Applied Biosystems), and reactions with 5 ng cDNA were carried out in an Eppendorf Mastercycler gradient thermal cycler (Sigma-Aldrich). The relative expression of osteogenic genes (*Alpl* and *Ibsp*), and a housekeeper gene (TATA-binding protein; *Tbp*) was determined using TaqMan Gene Expression assays (Applied Biosystems) as per the instructions of the manufacturer. Real-time PCR was performed using the QuantStudio 7 Flex real-time PCR machine (Applied Biosystems). Data is normalized to gene expression of cells cultured in OM at day 3. Relative quantification was determined using the 2^–ΔΔ^*^*CT*^* method.

#### Assessment of the Effect of Transfection With PEI-pIL-1Ra on BM-MSCs Cultured Under IL-1β-Driven Inflammatory Conditions

Having determined the dose-dependent effects of IL-1β on BM-MSC osteogenesis, we next sought to assess whether transfecting BM-MSCs with PEI-pIL-1Ra nanoparticles could render protection of their osteogenic phenotype while cultured under IL-1β-driven inflammatory conditions. BM-MSCs were cultured using osteogenic medium (OM), 1 ng/ml IL-1β alone or a combination of 1 ng/ml IL-1β and 10 ng/ml IL-1Ra. Additionally, BM-MSCs were transfected with either PEI-pGFP or PEI-pIL-1Ra nanoparticles, and cultured with OM supplemented with 1 ng/ml IL-1β. The expression of osteogenic markers (*Alpl* and *Ibsp*) in cells cultured under these conditions was evaluated after 3, 7 and 14 days, while mineral deposition was assessed from cells fixed with 4% paraformaldehyde (pH 7.4) after 21 days, and stained with 40 mM Alizarin Red S solution (pH 4.2) (Sigma Aldrich) for an hour at room temperature on a rotating plate.

### Development of a Novel Gene-Activated Scaffold Composed of PEI-pIL-1Ra Nanoparticles in Combination With a Collagen-Hydroxyapatite Scaffold for Bone Repair

#### Fabrication of Collagen-Hydroxyapatite Scaffolds

CHA scaffolds optimized for bone repair were fabricated as previously described (Cunniffe et al., 2010a; Curtin et al., 2012). Briefly, 1.8 g of bovine type I collagen (Southern Light Biomaterials, New Zealand) was added to 320 mL of 0.5 M acetic acid and blended at 15,000 rpm (Ultra Turrax T25 Overhead Blender, IKA Works, Inc., United States) for 90 min. 1.8 g (100 wt%) of hydroxyapatite nanoparticles, synthesized as previously described (Cunniffe et al., 2010b), was dissolved in 40 mL of 0.5 M acetic acid and added to the collagen slurry at a rate of 10 mL/h while blending at 15,000 rpm. The slurry is blended for a total time of 5 h. The slurry was degassed under a vacuum prior to lyophilization (Advantage EL, Vis-Tir, Co., Gardiner, NY, United States) to a final temperature of −40°C using a previously optimized lyophilization process (O’Brien et al., 2004; Haugh et al., 2010). The lyophilized scaffolds were physically cross-linked by dehydrothermal treatment of 105°C for 24 h at 0.05 bar in a vacuum oven (Vacucell 22, MMM, Germany), followed by chemical cross-linking using a mixture of 6 mM *N*-(3-Dimethylaminopropyl)-*N*′-ethylcarbodiimide hydrochloride (EDC) and 5.5 mM *N*-Hydroxysuccinimide (NHS) (Sigma-Aldrich). A biopsy punch (10 mm diameter) was used to cut scaffolds for subsequent experimental use.

#### Incorporation of PEI-pIL-1Ra Nanoparticles Into Collagen-Hydroxyapatite Scaffolds

CHA scaffolds were fabricated as described in Section “Fabrication of Collagen-Hydroxyapatite Scaffolds” and nanoparticles were formulated as described in Section “Complexation of pDNA to PEI.” Each scaffold was hydrated in PBS for 30 min before use and nanoparticles were incorporated via soak-loading.

#### Visualization of Gene-Activated Scaffolds Using Scanning Electron Micrography (SEM)

To visualize the nanoparticles on the porous surface of CHA scaffolds, 25 μL of the nanoparticle solution was added to each side of the scaffold. Samples were then dehydrated in 30 min stages from 30–50–70–90–95–100% ethanol. The samples were then flash-frozen in liquid nitrogen and freeze-dried. The samples were then mounted on metallic studs using carbon cement before being sputtered with gold/palladium alloy and imaged using SEM (Hitachi S-4700 II FESEM, Hitachi High Technologies, Germany). For comparison, the porous structure of CHA scaffolds, without nanoparticles, was also visualized.

#### Assessment of BM-MSCs Transfection in Gene-Activated Scaffolds

To assess the capacity of PEI-pIL-1Ra nanoparticles to transfect BM-MSCs on collagen-hydroxyapatite scaffolds, nanoparticles were formulated as described in Section “Complexation of pDNA to PEI.” After complexation, 25 μl of nanoparticle solution was loaded on each side of the scaffold, and after 10 min, 2.5 × 10^5^ BM-MSCs were loaded onto each side of the scaffold. After 24 h, the gene-activated scaffolds were moved to fresh 24-well plates in fresh expansion medium. Transfections in 3D were performed with PEI-pGLuc nanoparticles to quantify transgene expression up to day 21 on the scaffold, and with PEI-pIL-1Ra nanoparticles to visually assess transfection efficiency. The LIVE/DEAD Viability assay (Thermo Scientific) and Quant-iT^TM^ PicoGreen dsDNA assay (Invivogen, Switzerland) were performed 14 days post-transfection to evaluate potential nanoparticle-induced cytotoxicity in CHA scaffolds.

#### Assessment of the Effect of PEI-pIL-1Ra Nanoparticles on the Osteogenic Phenotype of BM-MSCs Cultured Under Inflammatory Conditions

BM-MSCs were cultured in CHA scaffolds using basal medium, OM or under inflammatory conditions (OM supplemented with 1 ng/ml IL-1β). Additionally, BM-MSCs were transfected in CHA scaffolds activated with PEI-pIL-1Ra nanoparticles and then cultured with OM supplemented with 1 ng/ml IL-1β. After 21 days of culture, scaffolds were then scanned using a CT scanner (40 kVp, voxel size 8 μm, VivaCT, Scanco, Switzerland) in order to visualize mineralization within the scaffold. 2D projection images were compiled to reconstruct 3D tomograms covering a volume within the scaffold with the shape of a cylinder with 4 mm diameter and 3 mm height using the standard Scanco software.

### Statistical Analysis

**FIGURE 1:**
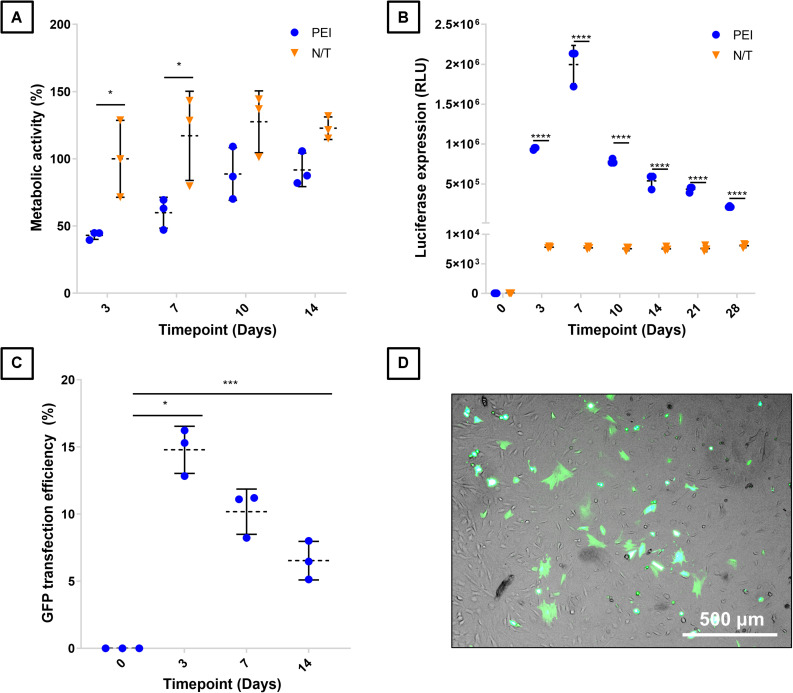
Characterization of BM-MSCs transfection with PEI-pDNA nanoparticles. BM-MSCs were transfected with PEI-GLuc and compared to non-transfected (N/T) in terms of **(A)** metabolic activity as a surrogate marker of cell viability, and **(B)** in terms of luciferase expression. BM-MSCs were also transfected with pGFP nanoparticles to determine their **(C)** transfection efficiency over time, while **(D)** brightfield and fluorescence microscopy image overlay shows GFP^+^, successfully transfected cells. *, ***, and **** denotes *p* < 0.05, 0.001, and 0.0001 respectively. Data plotted represents mean ± standard deviation, *n* = 3.

**FIGURE 2:**
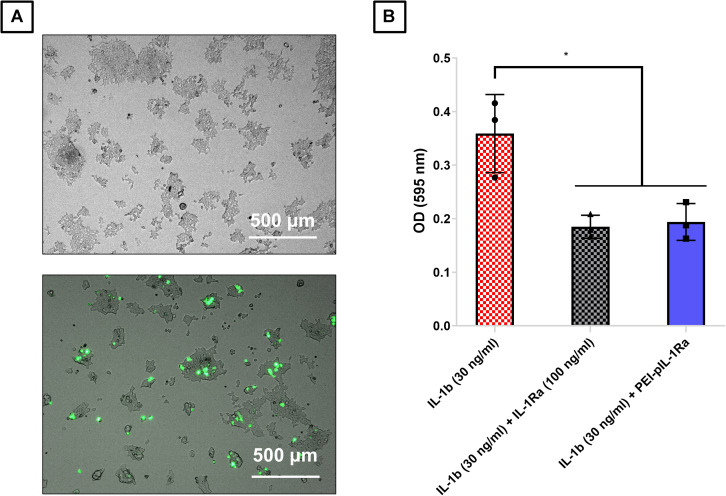
Assessment of PEI-pIL-1Ra nanoparticle gene product bioactivity. **(A)** Representative brightfield and fluorescence microscopy image overlay depicting non-transfected (top) and successfully transfected HEK-Blue IL-1β cells with PEI-pIL-1Ra nanoparticles (bottom). **(B)** Quantification of secreted enzymatic alkaline phosphatase activity in cells transfected with PEI-pIL-1Ra nanoparticles, in response to treatment with IL-1β (30 ng/ml), in comparison to non-transfected cells treated with IL-1β (30 ng/ml), and IL-1β (30 ng/ml) + IL-1Ra (100 ng/ml). * denotes *p* < 0.05. Data plotted represents mean ± standard deviation, *n* = 3.

**FIGURE 3:**
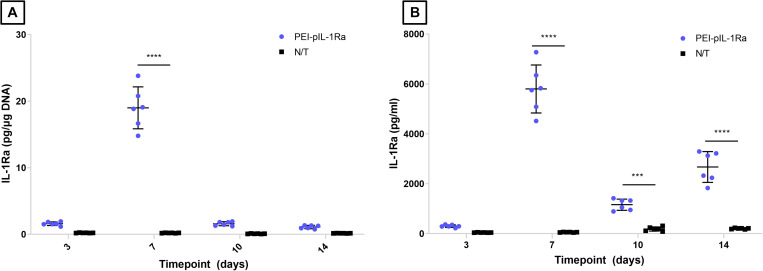
Assessment of IL-1Ra produced by rat BM-MSCs transfected with PEI-pIL-1Ra nanoparticles. Quantification of IL-1Ra produced by BM-MSCs, collected in culture media at 3, 7, 10, and 14 days post-transfection. **(A)** Data is normalized to μg of dsDNA present in cultures at each timepoint. **(B)** Data is expressed as pg/ml. *** and **** denotes *p* < 0.001 and *p* < 0.0001, respectively. Data plotted represents mean ± standard deviation, *n* = 3.

**FIGURE 4:**
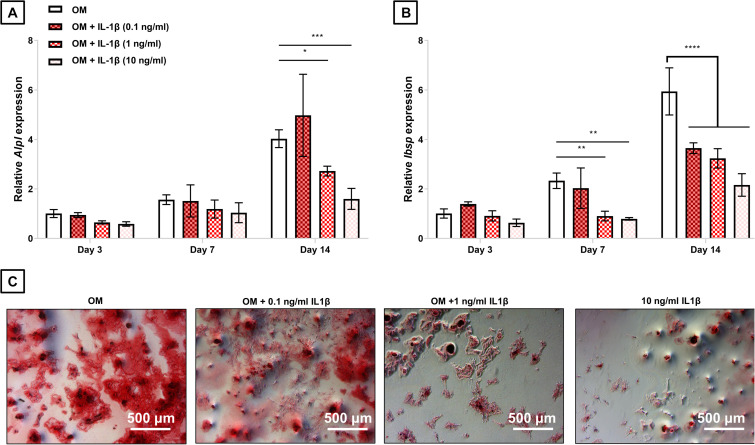
Effect of sustained IL-1β treatment on expression of osteogenic markers in rat BM-MSCs. Cells were cultured with osteogenic medium (OM) or OM treated with IL-1β (0.1, 1, and 10 ng/ml). The expression of **(A)** *Alpl* and **(B)** *Ibsp* were determined in response to these treatments after 3, 7, and 14 days in culture. **(C)** Mineral deposition in response to these treatments was assessed after 21 days. *, **, ***, and **** denotes *p* < 0.05, 0.01, 0.001, and 0.0001 respectively. Data is normalized to gene expression of cells cultured in OM at day 3. Relative quantification was determined using the 2^– ΔΔ^*^*CT*^* method. Data plotted represents mean ± standard deviation, *n* = 3.

**FIGURE 5:**
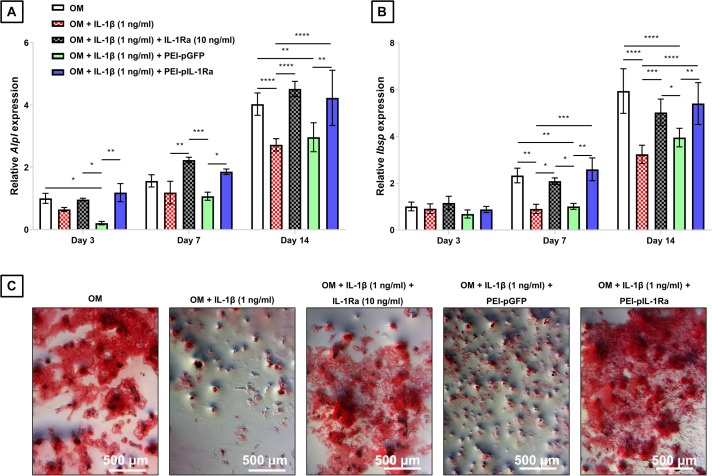
Effect of transfection with PEI-pIL-1Ra on rat BM-MSCs cultured in presence of IL-1β. Cells were transfected with either PEI-pGFP or PEI-pIL-1Ra and cultured with osteogenic media (OM) + IL-1β (1 ng/ml). For comparison, BM-MSCs were also cultured with OM, OM + IL-1β (1 ng/ml), and OM + IL-1β (1 ng/ml) + IL-1Ra (10 ng/ml). The effect of transfection with PEI-pIL-1Ra was assessed in terms of **(A)** *Alpl* gene expression, **(B)** *Ibsp* gene expression, and **(C)** mineral content deposition. *, **, ***, and **** denotes *p* < 0.05, 0.01, 0.001, and 0.0001 respectively. Data is normalized to gene expression of cells cultured in OM at day 3. Relative quantification was determined using the 2^– ΔΔ^*^*CT*^* method. Data shown represents mean ± standard deviation, *n* = 3.

Results are expressed as mean ± standard deviation. In Figures 1, 3–5, 7, a two-way ANOVA analysis was carried out followed by Bonferroni *post hoc* analysis, while in Figure 2, a *t*-test was carried out. For reproducibility, experiments were replicated at least three times (*n* = 3), unless stated otherwise, and where rat BM-MSCs were used, experiments were carried out using two donors purchased from Cyagen Biosciences (Jiangsu, China). *p* ≤ 0.05 values were considered statistically significant where ^∗^*p* < 0.05, ^∗∗^*p* < 0.01, ^∗∗∗^*p* < 0.001, and ^****^p < 0.0001.

**FIGURE 6:**
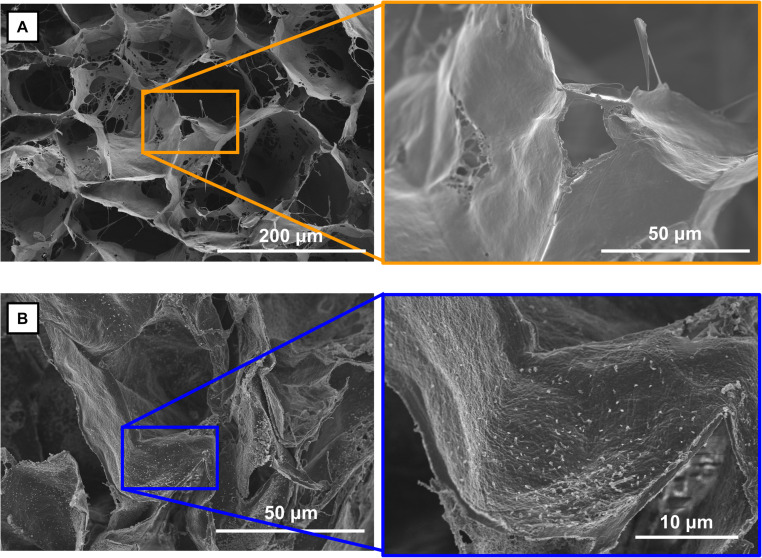
Visualization of PEI-pIL-1Ra nanoparticles in collagen-hydroxyapatite scaffold. **(A)** SEM image of the porous microarchitecture in CHA scaffolds, higher magnificent image shows smooth pore walls. **(B)** SEM image of a CHA scaffold activated with PEI-pIL-1Ra nanoparticles showing nanoparticles adhered to the porous structure.

**FIGURE 7:**
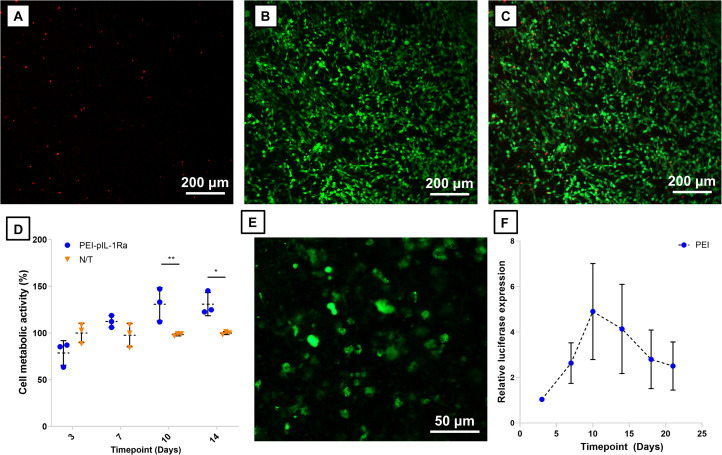
Assessment of BM-MSCs transfection in gene-activated CHA scaffolds. Cell viability of BM-MSCs in CHA scaffolds activated with PEI-pIL-1Ra nanoparticles after 3 days in culture is depicted in **(A–C)**: **(A)** Dead cells (ethidium homodimer 1-positive), **(B)** live cells (calcein-positive), **(C)** Overlay of live and dead cells. **(D)** Metabolic activity of cells in CHA scaffolds without nanoparticles (non-transfected) and CHA scaffolds activated with PEI-pIL-1Ra nanoparticles (PEI) after 3, 7, 10, and 14 days. **(E)** GFP^+^ cells were observed in CHA scaffolds activated with PEI-pIL-1Ra nanoparticles 3 days post-transfection. **(F)** The transgene expression profile of luciferase in CHA scaffolds activated with PEI-pGLuc nanoparticles, normalized to luciferase expression 3 days post-transfection. *, **, and *** denotes *p* < 0.05, *p* < 0.01, and *p* < 0.0001, respectively. Data plotted represents mean ± standard deviation, *n* = 3.

## Results

### Characterization of Rat BM-MSC Transfection With PEI-pIL-1Ra Nanoparticles

A characterization of BM-MSC transfection with PEI-pDNA nanoparticles was carried out using reporter genes pGFP and pGLuc. Transfection using PEI had an initial negative impact on cell viability, with a significant decrease in metabolic activity observed between non-transfected and transfected cells within 3 days of transfection (*p* < 0.05) (Figure 1A). However, the detrimental effect of PEI on metabolic activity was limited to early timepoints (<7 days), with a subsequent recovery to levels observed in non-transfected cells by 10 days post-transfection (Figure 1A). Transfection of BM-MSC with pGLuc revealed that a transient expression profile of the transgene is achieved in 2D, with a marked peak of expression recorded at 7 days post-transfection (Figure 1B). Fluorescent GFP^+^ cells were found interspersed throughout cultures of BM-MSC transfected with pGFP, indicating successful transfection using PEI (Figure 1C). Quantification of GFP^+^ cells within these cultures using flow cytometry revealed that the transfection efficiency was 14.8 ± 1.8% 3 days post-transfection (Figure 1D).

To assess the response of genes encoding for therapeutic protein, IL-1Ra, HEK-Blue IL-1β cells were transfected with PEI nanoparticles carrying pIL-1Ra. Cells were successfully transfected with PEI-pIL-1Ra nanoparticles, as indicated by the presence of GFP^+^ cells in transfected cultures, stemming from the presence of a GFP sequence in the pIL-1Ra plasmid (Figure 2A). A significant difference in IL-1β-mediated secreted enzymatic alkaline phosphatase (SEAP) levels was found between non-transfected HEK cells treated with IL-1β (30 ng/ml), and HEK cells transfected with PEI-pIL-1Ra treated with the same amount of IL-1β, which showed significantly lower levels of SEAP production (*p* < 0.05) (Figure 2B). This marked difference was equivalent to the difference found between non-transfected HEK cells treated with IL-1β (30 ng/ml), and non-transfected HEK cells treated with IL-1Ra (100 ng/ml) in addition to IL-1β (30 ng/ml) (*p* < 0.05) (Figure 2B).

The levels of IL-1Ra produced by rat BM-MSCs after transfection with PEI-pIL-1Ra nanoparticles were characterized. Non-transfected BM-MSCs were found to constitutively produce IL-1Ra at a rate of < 0.2 pg per μg of dsDNA. Transfection with PEI-pIL-1Ra led to a marked increase in IL-1Ra production. The IL-1Ra levels produced by transfected BM-MSCs were 8-, 95-, 8-, and 5-fold higher than non-transfected BM-MSCs, at 3, 7, 10, and 14 days after transfection, respectively (Figure 3).

### Assessment of the Capacity of IL-1Ra Gene Delivery to Protect the Osteogenic Phenotype of Rat BM-MSCs Cultured Under Inflammatory Conditions

Having confirmed the bioactivity of PEI-pIL-1Ra nanoparticles, and characterized the production of IL-1Ra by transfected BM-MSCs, we next sought to determine their potential therapeutic effects. First, we evaluated the effects of treatment with IL-1β on BM-MSC osteogenesis and found a dose-dependent negative impact of IL-1β on the gene expression of osteogenic markers *Alpl* and *Ibsp* (Figure 4). IL-1β at 1 ng/ml decreased the expression of *Alpl* after 14 days of culture under osteogenic medium (*p* < 0.05), while 10 ng/ml IL-1β led to an even greater reduction of *Alpl* expression at the same timepoint (*p* < 0.001) (Figure 4A). Both 1 and 10 ng/ml IL-1β were effective at suppressing the expression of *Ibsp* after only 7 days of culture under osteogenic medium (*p* < 0.01), while after 14 days, all IL-1β concentrations tested (0.1, 1, and 10 ng/ml) decreased *Ibsp* expression (*p* < 0.0001) (Figure 4B). After 21 days of culture, it was observed that treatment with increasing concentration of IL-1β led to markedly reduced mineral deposition (Figure 4C).

Having determined that 1 ng/ml was the minimum effective dose of IL-1β to significantly suppress the expression of osteogenic markers, we next sought to evaluate whether transfection with PEI-pIL-1Ra nanoparticles could protect BM-MSCs from IL-1β-mediated inhibition of osteogenesis. We found that transfecting BM-MSCs with PEI-pIL-1Ra was effective at protecting BM-MSCs from IL-1β-mediated inhibition of osteogenesis (Figure 5). No significant differences in *Alpl* and *Ibsp* expression were observed between BM-MSCs cultured in OM, and BM-MSCs transfected with PEI-pIL-1Ra cultured in OM + IL-1β. The expression of *Alpl* in BM-MSCs transfected with PEI-pIL-1Ra, cultured in OM + IL-1β, was significantly higher to the expression of *Alpl* in BM-MSCs cultured in OM + IL-1β after 14 days in culture (*p* < 0.0001) (Figure 5A). Similarly, the expression of *Ibsp* in BM-MSCs transfected with PEI-pIL-1Ra, cultured in OM + IL-1β, was significantly higher to the expression of *Ibsp* in BM-MSCs cultured in OM + IL-1β after 14 days in culture (*p* < 0.0001) (Figure 4B). The effect of transfection with PEI-pIL-1Ra on BM-BM-MSCs cultured in OM + IL-1β was equivalent to the effect of treating cells with recombinant IL-1Ra (10 ng/ml). The protective effect of transfection with PEI-pIL-1Ra on BM-MSCs cultured in OM + IL-1β was also derived from the expression of pIL-1Ra, and not from transfection alone, since treatment with PEI-pGFP nanoparticles did not confer a similar protective effect (Figure 5). In concert with the significant changes found in gene expression, we also observed marked changes in the phenotype of BM-MSCs in terms of mineral deposition (Figure 5C). After 21 days of culture, BM-MSCs cultured in OM appeared to have similar levels of mineral deposition compared to BM-MSCs transfected with PEI-pIL-1Ra nanoparticles cultured in OM + IL-1β, while non-transfected BM-MSCs cultured in OM + IL-1β and BM-MSCs transfected with PEI-pGFP cultured in OM + IL-1β showed markedly less mineral deposition (Figure 5C).

### Incorporation of PEI-pIL-1Ra Nanoparticles Into Collagen-Hydroxyapatite Scaffolds

Having characterized the transfection of BM-MSCs with PEI-pDNA nanoparticles, and having determined the therapeutic effects of PEI-pIL-1Ra nanoparticles on BM-MSCs cultured in the presence of IL-1β, we next sought to establish whether this non-viral gene therapy could be deployed in CHA scaffolds. SEM analysis following loading of PEI-pIL-1Ra nanoparticles revealed that the nanoparticles were localized and bound to the struts and walls of the porous microarchitecture in CHA scaffolds (Figure 6). The smooth texture of the microarchitecture in CHA scaffolds (Figure 6A) was contrasting in comparison to the microarchitecture in CHA scaffolds loaded with PEI-pIL-1Ra nanoparticles, which appeared to be littered with sub-micron sized particles (Figure 6B).

Having observed a limited cytotoxic effect of PEI treatment on BM-MSCs in 2D culture, we sought to determine whether transfection of BM-MSCs in CHA scaffolds rendered them less susceptible to cytotoxic effects, either by virtue of the 3D culture environment or through reduced exposure to high concentrations of nanoparticles in 2D cultures. Very few dead cells were found in CHA scaffolds activated with PEI-pIL-1Ra nanoparticles 3 days post-transfection (Figure 7A), while > 99% of cells appeared viable (Figures 7B,C). After transfection, a significant decrease (*p* < 0.01) of metabolic activity was observed in cells cultured in gene activated CHA scaffolds after 3 days (Figure 7D). However, this effect was found to be acute as there was a significant increase (*p* < 0.01) in metabolic activity at 10 and 14 days post-transfection (Figure 7D). Transfection of BM-MSCs with PEI-pIL-1Ra nanoparticles was successful in CHA scaffolds, indicated by the presence of bright GFP^+^ cells 3 days post-transfection (Figure 7E), while activation of CHA scaffolds with PEI-pGLuc nanoparticles confirmed that the transgene expression profile was retained in 3D (Figure 7F).

**FIGURE 8:**
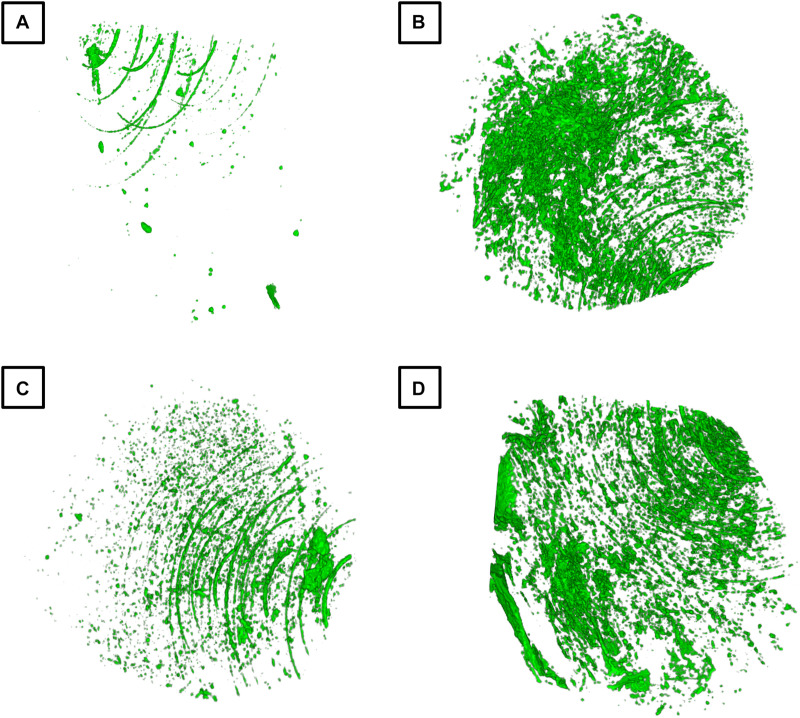
Protective effect of PEI-pIL-1Ra nanoparticles on IL-1β-mediated inhibition of osteogenesis in BM-MSCs. Representative 3D microCT reconstructions depicting mineral deposition by BM-MSCs after 21 days in CHA scaffolds cultured under **(A)** basal medium, **(B)** osteogenic medium, **(C)** osteogenic medium + IL-1β (1 ng/ml), and **(D)** osteogenic medium + IL-1β (1 ng/ml) + PEI-pIL-1Ra nanoparticles. Diameter of each cylindrical reconstruction is 4 mm, while height is 3 mm.

Finally, we sought to determine whether osteogenic differentiation within CHA scaffolds was inhibited by IL-1β treatment (1 ng/ml), and whether this inhibitory effect could then be mitigated in CHA scaffolds incorporating PEI-pIL-1Ra (Figure 8). We found that the minimum effective dose of IL-1β (1 ng/ml) that effectively suppressed osteogenesis in 2D was also effective at markedly inhibiting mineralized matrix production by BM-MSCs in the 3D environment of CHA scaffolds (Figure 8). OM was effective at inducing mineral deposition in CHA scaffolds, with an average mineral volume: total volume (MV/TV) ratio of 0.767% (Figure 8B) in contrast to basal medium, which had a MV/TV of 0.053% (Figure 8A). Sustained treatment with IL-1β in OM effectively suppressed this osteogenic phenotype, reducing the MV/TV to 0.162% (Figure 8C). Supporting our primary hypothesis, this inhibitory effect could then be mitigated in scaffolds incorporating PEI-pIL-1Ra nanoparticles, which had an average MV/TV of 0.913% (Figure 8D).

## Discussion

As a result of our increasing understanding of the immune system’s role in regulating fracture healing, there is increasing demand for novel therapies that can not only support new bone formation, but also immunomodulate the local fracture microenvironment. In this study, a novel gene-activated scaffold was developed, and assessed *in vitro*, by incorporating non-viral PEI-pDNA nanoparticles encoding for IL-1Ra into CHA scaffolds, which have proven capacity to support bone repair (Gleeson et al., 2010). This platform demonstrated the capacity to effectively delivery the gene encoding for IL-1Ra to rat BM-MSCs, conferring protection to their osteogenic phenotype under IL-1β-mediated inflammatory conditions, both in 2D and in the 3D environment of CHA scaffolds. This development marks a promising approach for applications in long bone repair, where non-viral gene therapy could effectively be used to immunomodulate the local microenvironment and potentially make it more permissive for healing.

While the CHA scaffold used in this study is a proven platform for the local delivery of non-viral gene therapy for bone repair (Curtin et al., 2015; Raftery et al., 2015, 2016), its use as a platform specifically for the delivery of immunomodulatory genes with prospective applications in long bone repair is a major novelty. Despite modern orthopedic fixation technologies, which work by facilitating bone’s intrinsic capacity to heal after injury, large bone defects arising from tumor resections, or complex, comminuted fractures, represent a challenging pathology that can result in delayed healing and non-union (Santolini et al., 2015). Prolonged inflammation at fracture sites, lasting longer than initially required to support the establishment of a fracture hematoma, is recognized as an important factor that impinges on the fracture healing process and contributes directly to subsequent healing complications (Guo et al., 2008; Reinke et al., 2013; Santolini et al., 2015; Sun et al., 2017). The CHA scaffold used in our study has proven efficacy as a standalone treatment for bone repair, as evaluated in a rat calvarial defect model, and is clinically used in maxillofacial applications (Cunniffe et al., 2010a; Gleeson et al., 2010; Curtin et al., 2012; Castano et al., 2015). However, the calvarial defect is essentially non-weight bearing and carries with it a diminished inflammatory response to that which would be typically observed after a long bone fracture and the onset of callus formation driven by local micromotion due to mechanical loading. Thus, using the collagen-hydroxyapatite scaffold as a platform for the delivery of immunomodulatory genes, such as IL-1Ra, is both highly warranted and has great clinical potential.

In this study, non-viral gene delivery of IL-1Ra proved to be effective at conferring protection to rat BM-MSCs against suppression of osteogenesis inferred by sustained IL-1β treatment *in vitro*. This effect was equivalent to sustained treatment with recombinant IL-1Ra, highlighting the versatility of non-viral viral vectors, and their transient, but sustained therapeutic capacity. This property of non-viral gene therapy is highly advantageous in comparison to a recombinant protein-based therapy, which would require much higher doses or even repeated administration of doses to achieve the same effect as biomaterial-based delivery typically exhibit burst-release of their drug cargos (Quinlan et al., 2017). The sustained effect of non-viral gene therapy also counteracts the limited transfection efficiency of some non-viral gene delivery vectors. In this study, a low transfection efficiency of 15% was observed with PEI, which might raise concerns of a limited therapeutic effect. However, despite a low transfection efficiency, we were able to observe a transient bioactivity, which was able to counteract the potent effects of sustained IL-1β treatment. This capacity of non-viral gene therapy, synonymous with ‘less is better,’ has previously been observed while using PEI to deliver angiogenic genes (Curtin et al., 2015), osteogenic genes (Raftery et al., 2017), and neurotrophic genes (Lackington et al., 2018) for a variety of applications.

The choice of PEI in this study was due to its proven potential in 2D and 3D scaffold applications in our team (Tierney et al., 2012; Curtin et al., 2015; Lackington et al., 2018), although alternatives including RALA (Bennett et al., 2015), chitosan (Raftery et al., 2015), poly(L-lysine) (Walsh et al., 2018) and nanohydroxyapatite (Castano et al., 2015), might present an improved choice of non-viral gene delivery vector for both efficiency of transfection and/or decreased cytotoxicity. The detrimental cytotoxic effects of PEI observed in 2D at early time-points may be avoided by using another vector, while the vector choice presents an interesting opportunity to further immunomodulate a fracture microenvironment. It has been reported that BM-MSC fate following non-viral gene transfection can be strongly manipulated by the choice of delivery vector (Gonzalez-Fernandez et al., 2017). Similarly, it is likely that different non-viral gene delivery vectors have different propensities for stimulating a pro- or anti-inflammatory response, which might enhance or suppress the effect of their therapeutic cargo. This is supported by a recent study reporting that the size and shape of hydroxyapatite particles can be tuned to stimulate M2 macrophage polarization (Mahon et al., 2020). A limited assessment might be possible to determine the effect of different non-viral gene delivery vectors on M1/M2 macrophage polarization; however, *in vivo* assessment would ultimately be required to better understand the relationship between non-viral gene delivery vectors and the immune response that they trigger.

The novel PEI-pIL-1Ra nanoparticles developed in this study were seamlessly incorporated into collagen-hydroxyapatite scaffolds, maintaining their capacity to transfect rat BM-MSCs in 3D, exhibiting sustained but transient gene expression *in vitro*. Supporting our primary hypothesis, mineralization induced by osteogenic medium within scaffolds could be inhibited by chronic exposure to IL-1β, and it was then demonstrated that this inhibitory effect could then be mitigated in scaffolds incorporating PEI-pIL-1Ra nanoparticles, as desired. The next step in the validation of this approach should involve *in vivo* assessment using an inflammatory, long bone fracture model to determine the level to which this non-viral gene therapy can enhance bone repair.

## Conclusion

In this study, a novel immunomodulatory gene-activated scaffold, consisting of a collagen-hydroxyapatite scaffold loaded with PEI-pIL-1Ra nanoparticles, was developed. The transient nature of therapeutic gene expression in our approach potentially enables the preservation of the initial pro-inflammatory response to fracture, which is crucial to the healing cascade. This offers a key advantage over other immunomodulatory approaches, including recombinant protein and viral gene therapies, which have less temporal control compared to non-viral gene-based therapies.

## Data Availability Statement

The raw data supporting the conclusions of this article will be made available by the authors, without undue reservation.

## Author Contributions

WL and MG-S conducted experiments, acquired data, and performed data analysis. WL and KT designed the study and wrote the manuscript. AG-V fabricated biomaterials. AG-V, FO’B, and MS critically revised the manuscript. All authors have read and approved the submitted version of the manuscript.

## Conflict of Interest

The authors declare that the research was conducted in the absence of any commercial or financial relationships that could be construed as a potential conflict of interest.
